# Comparison of Post-operative Outcomes Between Direct Axillary Artery Cannulation and Side-Graft Axillary Artery Cannulation in Cardiac Surgery: A Systematic Review and Meta-Analysis

**DOI:** 10.3389/fcvm.2022.925709

**Published:** 2022-06-10

**Authors:** Yi Xie, Yu Liu, Peng Yang, Chen Lu, Jia Hu

**Affiliations:** ^1^Department of Cardiovascular Surgery, West China Hospital, Sichuan University, Chengdu, China; ^2^Department of Cardiovascular Surgery, West China Guang'an Hospital, Sichuan University, Guang'an, China

**Keywords:** axillary artery cannulation, direct cannulation, side-graft cannulation, neurological dysfunction, cardiopulmonary bypass

## Abstract

**Background:**

There is a growing perception of using axillary artery cannulation to improve operative outcomes in cardiopulmonary bypass surgery. Two techniques, direct cannulation or side-graft cannulation, can be used for axillary artery cannulation, but which technique is better is controversial.

**Methods:**

A meta-analysis of comparative studies reporting operative outcomes using direct cannulation vs. side-graft cannulation was performed. We searched the PubMed, EMbase, Web of Science, and Cochrane Library. Outcomes of interest were neurological dysfunction, cannulation-related complications and early mortality. The fixed effects model was used.

**Results:**

A total of 1,543 patients were included in the final analysis. Direct cannulation was used in 846 patients, and side-graft cannulation was used in 697 patients. Meta-analysis showed a higher occurrence of neurological Complication in direct cannulation group [odds ratio, 1.45, 95% CI (1.00, 2.10), χ^2^ = 4.40, *P* = 0.05] and a significantly higher incidence of cannulation-related complications in the direct cannulation group [odds ratio, 3.12, 95% CI (1.87, 5.18), χ^2^ = 2.54, *P* < 0.0001]. The incidence of early mortality did not have a difference [odds ratio, 0.95, 95% CI (0.64, 1.41), χ^2^ = 6.35, *P* = 0.79].

**Conclusions:**

This study suggests that side-graft axillary artery cannulation is a better strategy as it reduces the incidence of neurological dysfunction and cannulation-related complications.

**Systematic Review Registration:**

https://www.crd.york.ac.uk/PROSPERO/, identifier: CRD42022325456.

## Introduction

Neurological dysfunction (1–3) [permanent neurological dysfunction (4) or contemporary neurological dysfunction] is a major morbidity and mortality after cardiopulmonary bypass (CPB). In addition, the incidence of stroke after cardiac surgery is ~6% (5), not only affecting the length of hospital stay to increase the cost of treatment, but also having an effect on the quality of life (6, 7). The released atherosclerotic plaques cause embolism and the insufficiency of cerebral perfusion can lead to neurological dysfunction during CPB (8, 9). As a consequence, it is essential for cardiac surgeons to choose right the cannulation method, especially when hypothermic arrest for aortic surgery, it is essential to choose right cerebral protection to decrease the incidence of neurological dysfunction in CPB (10).

In addition, for axillary artery cannulation, we may also concern about cannulation-related complications which influence the recovery of patients, even result in devasting results. Also, axillary artery cannulation methods can be a factor influencing the post-operative mortality for cardiac surgery.

Although the best arterial cannulation site is still unknown, a growing body of data suggests the good outcomes of axillary artery (AX) cannulation in aortic surgery and complex cardiac surgery (11–13). Because femoral artery cannulation with retrograde cerebral perfusion has the potential to induce brain embolization or organ perfusion (14, 15), especially in patients with severe atherosclerosis. Also, previous studies have shown the superiority of axillary artery cannulation (16, 17) in reducing the incidence of postoperative neurological dysfunction and survival outcomes, which can preserve antegrade blood flow.

The method of axillary artery cannulation, including direct cannulation or side-graft cannulation (Ax+SG), has been used universally (18, 19). Many institutions at first may use direct cannulation for its expediency and less bleeding during CPB. However, axillary artery may be damaged due to its fragility resulting in a higher incidence of complications, such as the dissection of axillary artery (20) or axillary artery narrowing (21). Arm ischemia (22) is also a major concern, which is devasting for patients. Although there are some observational studies comparing direct cannulation and side-graft cannulation, the neurological and survival outcomes are conflicting. Moreover, without randomized controlled studies to compare these two techniques, and direct cannulation is still preferred by cardiac surgeons during CPB. Thus, we conducted a meta-analysis of investigation dealing with direct or side-graft cannulation during CPB.

## Materials and Methods

### Literature Search and Selection of Articles

The literature research to confirm eligible studies was conducted by two trained reviewers (Yi Xie and Peng Yang) independently comparing direct axillary cannulation vs. side-graft axillary cannulation on the incidence of neurological deficits. PubMed, EMbase, Web of Science, and Cochrane Library were searched up to date of 1 Noverber 2021. The research strategy: (“cardiopulmonary bypass” [MeSH Terms] OR “heart lung bypass” [Title/Abstract] OR “bypass heart lung” [Title/Abstract] OR ((“Bypass” [All Fields] OR “bypassed” [All Fields] OR “Bypasses” [All Fields] OR “bypassing” [All Fields]) AND “Heart-Lung” [Title/Abstract]) OR “heart lung bypass” [Title/Abstract] OR ((“Heart-Lung” [Journal] OR (“Heart” [All Fields] AND “Lung” [All Fields]) OR “Heart-Lung” [All Fields]) AND “Bypasses” [Title/Abstract]) OR “bypass cardiopulmonary” [Title/Abstract] OR “cardiopulmonary bypasses” [Title/Abstract] OR “cardiac” [Title/Abstract] OR “aortic” [Title/Abstract]) AND (“axillary artery” [MeSH Terms] OR “arteries axillary” [Title/Abstract] OR “artery axillary” [Title/Abstract] OR “axillary arteries” [Title/Abstract]) AND (“cannulation” [Title/Abstract] OR “direct” [Title/Abstract] OR “graft” [Title/Abstract] OR “prothesis” [Title/Abstract]). In addition, we manually researched the reference list of the studies to include other eligible studies. This process was repeated until no any new study was found. Finally, all included studies were unanimously approved. We also defined the PICOS guidelines in this study. P: patients undergoing cardiac surgery with axillary artery cannulation during cardiopulmonary bypass; I: side-graft cannulation; C: direct cannulation; O: primary outcomes with neurological dysfunction and cannulation-related complications, secondary outcome with in-short mortality; S: observation studies.

### Eligibility and Exclusion Criteria

The inclusion criteria were as follows: (i) involved cannulation of direct axillary cannulation and side-graft axillary cannulation in patients with cardiopulmonary bypass; (ii) included at least one primary outcome; (iii) study included at least 10 serial patients to prevent bias in a small population. The exclusion criteria were: (i) <10 patients for case reports and case series; (ii) study cannot extract primary outcome; (iii) axillary artery cannulation techniques [direct cannulation or side-graft] cannot be defined]; (iv) Studies that have not reported or could not be calculated comparing two canulation techniques from published results.

### Data Extraction and Outcomes

Two reviewers (YiXie and Peng Yang) performed data extraction and the following characteristics including first author, publication year of study, patient baseline characteristics, sample size, study design, direct cannulation group intervention, control group intervention were extracted; for the patient sample: patient gender, mean age, preoperative comorbidities. The intraoperative data included cerebral perfusion time and CPB time. The post-operative outcome: neurological dysfunction (3) (permanent neurological dysfunction and transient neurological dysfunction), cannulation-related complications, early mortality (in-hospital or 30-day mortality), and length of hospital stay (LOHS). In addition, a third reviewer (Chen Lu) reevaluated the extracted data and all disputes were resolved by consensus. The primary results were the rate of neurological dysfunction and complications related to cannulation. Secondary outcome was early mortality. Neurological dysfunction included permanent and contemporary neurological dysfunction. Cannulation-related complications (21, 23) included brachial plexus injury, iatrogenic axillary artery dissection, aortic dissection, axillary artery thrombosis, high perfusion resistance, hyperperfusion syndrome, malperfusion, surgical repair and arm ischemia. Early-mortality was defined as in-hospital or 30-day mortality.

### Quality Assessment

The study was performed comply with the recommendations of the proposal for reporting meta-analysis of observational studies in epidemiology (32). The quality of the observations studies was assessed by using Newcastle–Ottawa Scale. The quality of the studies was evaluated by examining three items: patient selection, comparability of direct cannulation and Ax+SG groups, and assessment of outcomes. For the comparability between the two groups, we focused on the following variables: age, sex, surgical procedures, perioperative comorbidities. If discrepant results were obtained, the articles were re-analyzed by the two reviewers and a consensus was reached.

### Statistical Analyses

Odd ratios (ORs) and their 95% confidence intervals (CIs) were used to assess the difference between the two groups. Heterogeneity among studies was estimated using the χ2 test and the Cochran Q score (reported as *I*^2^ and representing the percent value of the heterogeneity). 25%-50% *I*^2^ statistics indicate low heterogeneity, medium (50%-75% *I*^2^ statistics) or high (*I*^2^ statistics >75%). Q-statistic and *I*^2^ statistics determine the models: for *I*^2^ <50% and heterogeneity *P* > 0.10, a fixed effects model was used; Otherwise, we adapt a random-effects model. Review Manager Version 5.4 (The Cochrane Collaboration, Software Update, Oxford, UK.) was used to perform all statistical analyses. A two-tailed *P*-value of <0.05 indicates statistical significance of meta-analysis. Unless, a *P*-value for significance was specifically declared. Subgroup analyses were performed for patients who underwent only thoracic aortic surgery. We also used leave-one out analysis for sensitivity.Publication bias assessment was analyzed by funnel plots.

## Results

### Quantity of Evidence

**Figure 1:**
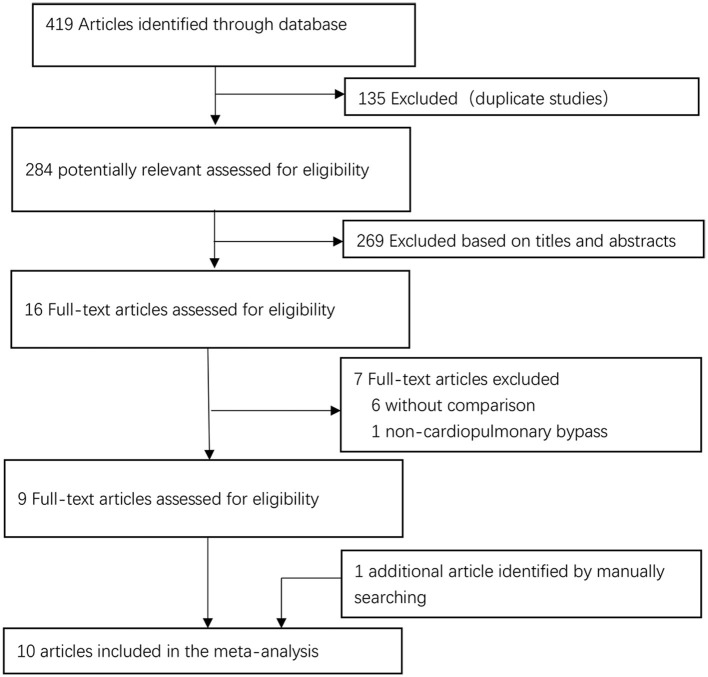
Study selection flow diagram.

The literature search yielded 419 publications. Of these studies, 135 were excluded because they showed duplicate ones, and 269 were excluded after initial screening of titles and abstracts. The remaining 16 studies were further evaluated. Among the remained articles, seven publications were excluded for further assessment of full text without comparison or non-cardiopulmonary bypass. Additionally, we also retrieved a study by manual search of reference lists. Thus, a total of ten studies meeting the inclusion criteria were finally included in the meta-analysis (Figure 1).

### Qualities of Results

Ten included studies were assessed the quality by Newcastle–Ottawa Scale. Table 1 indicates further details of these studies with quality assessment. The study period ranged from 1993 to 2021. All included studies reported on neurological dysfunction, and 7 studies reported on the incidence of cannulation-related complications. Seven of ten studies showed a high-level quality.

**Table 1:** Newcastle-Ottawa quality assessment of ten eligible studies.

**Studies (year)**	**selection**				**Comparability of cohorts**	**Outcome**			**Score**
	**Representa tiveness of exposed cohort**	**Selection of non-exposed cohort**	**Ascertainment of exposure**	**Outcome of interest not present at start of study**		**Assessment of outcome**	**Follow-up long enough for outcome**	**Adequacy of follow up**	
Puiu et al. (23)	^⋆^	^⋆^	^⋆^	^⋆^	^⋆⋆^	^⋆^	^⋆^	^⋆^	9
Talwar et al. (24)		^⋆^	^⋆^	^⋆^	^⋆^	^⋆^	^⋆^	^⋆^	7
Jia et al. (25)	^⋆^	^⋆^	^⋆^	^⋆^	^⋆^	^⋆^	^⋆^	^⋆^	8
Do et al. (26)		^⋆^	^⋆^	^⋆^	^⋆^	^⋆^	^⋆^	^⋆^	7
Yilik et al. (27)		^⋆^	^⋆^	^⋆^	^⋆^	^⋆^	^⋆^	^⋆^	7
Mastroroberto et al. (28)		^⋆^	^⋆^	^⋆^		^⋆^	^⋆^		5
Kokotsakit et al. (29)			^⋆^	^⋆^	^⋆^	^⋆^	^⋆^	^⋆^	6
Fleck et al. (30)		^⋆^	^⋆^	^⋆^	^⋆^	^⋆^	^⋆^	^⋆^	7
Sabik et al. (21)		^⋆^	^⋆^	^⋆^	^⋆⋆^	^⋆^	^⋆^	^⋆^	8
Schachner et al. (31)		^⋆^	^⋆^	^⋆^	^⋆^	^⋆^		^⋆^	6

⋆ *: 1*;

⋆⋆ *: 2*.

### Basic Demographics

The basic characteristics are presented Table 2 including demographic features and cardiopulmonary details. Post-operative outcomes are presented in Table 3. For included studies, most of patients undergone aortic surgery or reoperation with axillary artery cannulation.

**Table 2:** Baseline characteristics of the ten eligible articles.

**Author(year)**	**Design**	**Years**	**N**	**Age**		**Male (%)**	**Surgical type**	**Comorbidities**	**CPB (min)**	
				**Direct**	**Ax+SG**				**Direct**	**Side-graft**
Puiu et al. (23)	R	2008–2019	532	66(55–75)	67(56–74)	204	a	1–8	174.0(139.0–215.0)	192.5(150.0–231.0)
Talwar et al. (24)	R	2013–2017	68	63.9	ND	a	NA	NA	NA
Jia et al. (25)	R	2008–2010	328	43.1 ± 9.5	44.7 ± 8.3	199	a	1,2,4,6	145.0 ± 26.8	152.4 ± 23.5
Do et al. (26)	R	2003–2009	53	54.8 ± 14.2	56.7 ± 13.4	26	a	1,7,8	194.0 ± 40.8	164.1 ± 51.4
Yilik et al. (27)	P	2001–2004	68	49.3 ± 11.2	53.4 ± 10.5	45	a	1–4,6–8	NA	NA
Mastroroberto et al. (28)	R	1999–2004	26	58–77	21	a/b	NA	NA	NA
Kokotsakit et al. (29)	R	2000–2004	27	64	20	a/b	NA	NA	NA
Fleck et al. (30)	R	2002–2004	70	62.9 ± 12.3	58.1 ± 12.5	53	a	NA	147.5 ± 55.8	165.7 ± 69.2
Sabik et al. (21)	R	1993–2001	399	67 ± 12	67 ± 13	155	a/b	1,3,4,6,8	147(118–192)	147(124–186)
Schachner et al. (31)	R	2000–2002	22	63(22–27)	17	a	NA	NA	NA

*R, retrospective; P, prospective; NA, not available; N, number of patients; surgical procedure: a, thoracic aortic surgery; b, mixed cardiac surgery (CABG, valve surgery et al.); comorbidities: 1, hypertension; 2, coronary artery disease; 3, COPD; 4, peripheral artery occlusive disease; 5, neurological deficit; 6, diabetes mellitus; 7, marfan syndrome; 8, malperfusion (cerebral, visceral and lower extremity); CPB: cardiopulmonary bypass*.

**Table 3:** Outcomes of ten eligible studies.

**Author (year)**	**No. of patients**		**Neurological dysfunction**		**Cannulation-related complications**		**Early mortality**		**LOHS (d)**	
	**Direct**	**Ax+SG**	**Direct**	**Ax+SG**	**Direct**	**Ax+SG**	**Direct**	**Ax+SG**	**Direct**	**Ax+SG**
Puiu et al. (23)	266	266	41	24	33	15	22	24	17.0 (13.0–23.0)	16.0 (12.0–22.0)
Talwar et al. (24)	29	39	2	5	NA	NA	8	4	13 ± 8.02	16 ± 11.02
Jia et al. (25)	215	65	19	6	19	1	7	3	8.6 ± 3.4	9.3 ± 2.8
Do et al. (26)	18	35	4	9	1	1	0	3	ND	NA
Yilik et al. (27)	22	46	1	1	3	0	4	5	8.3 ± 2.2	8.0 ± 4.5
Mastroroberto et al. (28)	21	5	0	0	NA	NA	NA	NA	NA	NA
Kokotsakit et al. (29)	4	23	0	1	0	0	0	1	NA	NA
Fleck et al. (30)	46	24	4	0	NA	NA	NA	NA	9.95 ± 4.2	8.4 ± 3.8
Sabik et al. (21)	212	187	13	8	16	4	12	16	NA	NA
Schachner et al. (31)	13	7	0	0	0	0	NA	NA	11 (4-66)

*No. of patients: number of patients; Ax + SG: side-graft axillary artery cannulation; LOHS: length of hospital stay; NA: not available*.

### Primary Outcome

**Figure 2:**
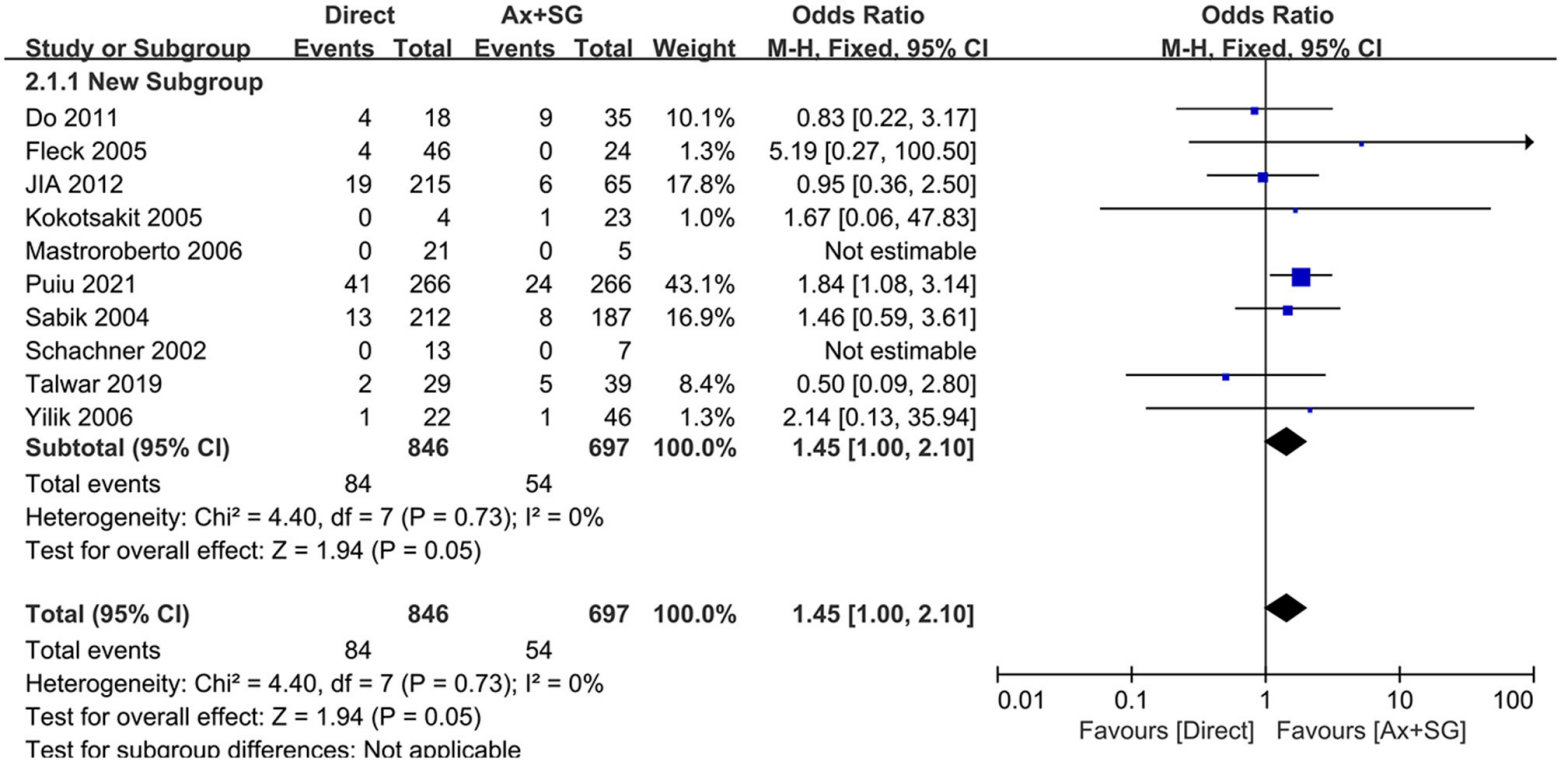
Neurological dysfunction in direct artery cannulation vs. side-graft artery cannulation.

Neurological dysfunction was reported in ten studies, and A meta-analysis of data revealed that the pooled mortality incidence was 10.0% malperfusion, compared to 7.6% in the Ax+SG group. Figure 2 shows a difference in neurological dysfunction reaching statistical significance [odds ratio, 1.45, 95% CI (1.00, 2.10), χ^2^ = 4.40, *P* = 0.05]. The test for heterogeneity was low and not statistically significant (*I*^2^ = 0; *P* = 0.73), which revealed the validity of the pooling data.

**Figure 3:**
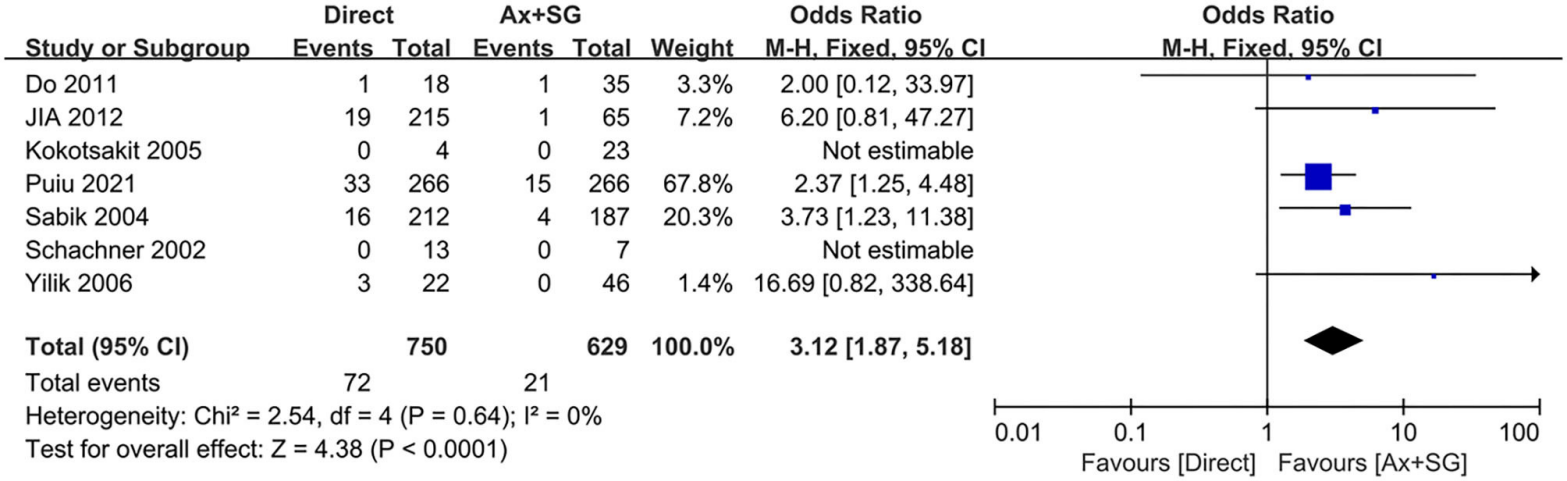
Cannulation-related complication in direct artery cannulation vs. side-graft artery cannulation.

Cannulation-related complications were reported in seven studies. The pooled cannulation-related complications rate in the direct cannulation group was 9.6% when compared with 3.3% in the Ax+SG group. As shown in Figure 3, differences of cannulation-related complications reach statistical significance [odds ratio, 3.12, 95% CI (1.87, 5.18), χ^2^ = 2.54, *P* < 0.0001]. The test for heterogeneity was low and not statistically significant (*I*^2^ = 0; *P* = 0.64), which indicates the validity of the pooling data.

### Secondary Outcome

**Figure 4:**
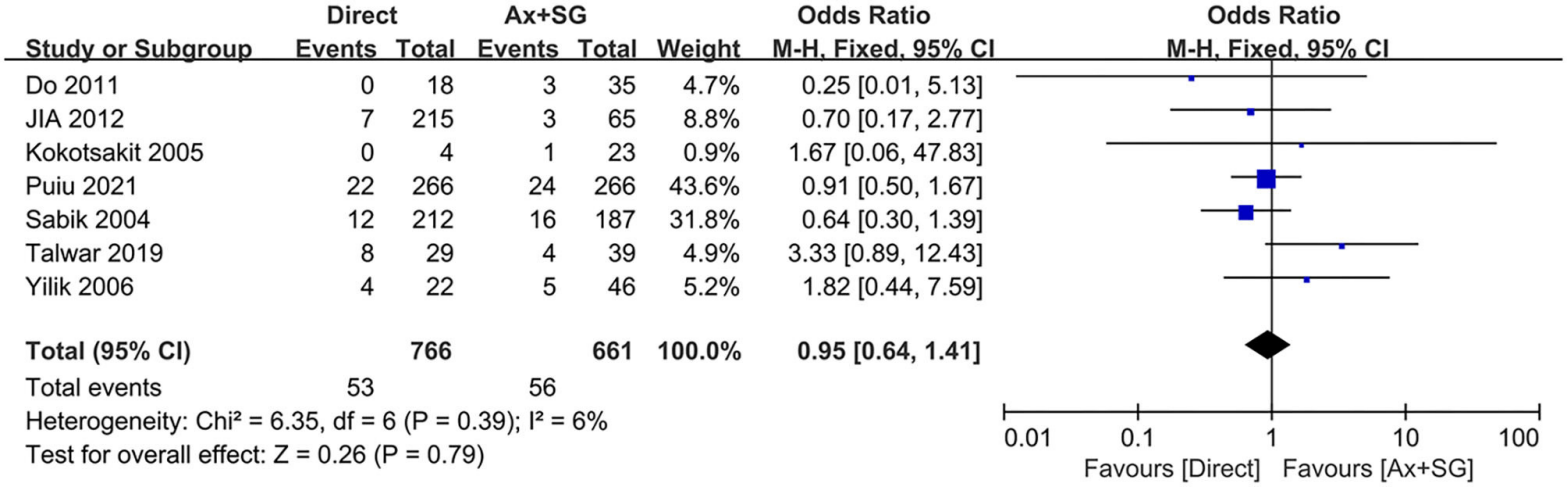
Post-operative mortality in direct artery cannulation vs. side-graft artery cannulation.

The difference in postoperative mortality was extracted from 7 studies. The pooled postoperative mortality incidence between direct cannulation group was 6.9%, which was not statistically significant compared to 8.5% in the SGAC group [probability ratio, 0.95, 95% CI (0.64, 1.41), χ^2^ = 6.35, *P* = 0.79], shown in Figure 4. The test of heterogeneity was not statistically significant (*I*^2^ = 6%, *P* = 0.39).

### Sensitivity and Publication Bias Analysis

**Figure 5:**
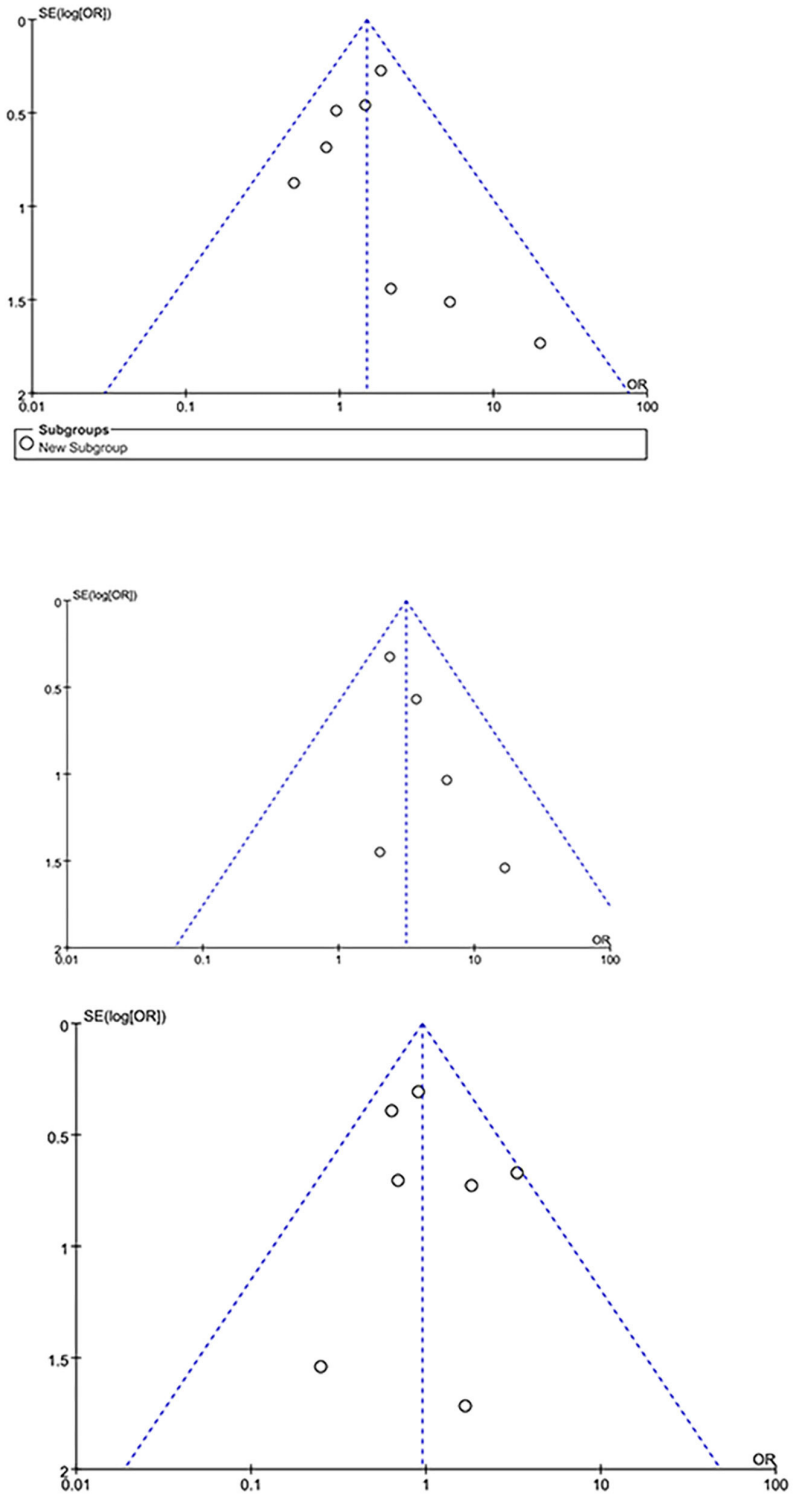
The funnel plot for neurological dysfunction (above), cannulation-related complications (middle) and early mortality (below).

For subgroup analysis, studies only reporting patients undergoing aortic surgery (Table 1) were pooled, no significant difference was observed in neurological dysfunction between both group [(odds ratio, 1.44; 95% CI, (0.95–2.17); *P* = 0.08; *I*^2^ = 0)]. For leave-one out analysis for sensitivity analysis with primary outcomes of our study, as is shown in Tables 4, 5, the heterogeneity is low and the pooled results are robust. The publication bias was evaluated by funnel plot. Neurological dysfunction, cannulation-related complications and early mortality outcome was shown in Figure 5. For cannulation-related complications and early mortality, the plot appeared symmetrical, indicating that no publication bias has occurred in the study. However, for neurological dysfunction, the plot appeared asymmetrical, indicating some publication bias.

**Table 4:** Sensitivity with leave-one out analysis for neurological analysis.

**Leave-one out analysis**	**OR, 95%CI**	**χ^2^ (Ch*i2*)**	***I*** **^2^**	***P*-value for heterogeneity**
Do et al. (26)	1.52 [1.03, 2.24]	3.70	0%	0.72
Fleck et al. (30)	1.40 [0.96, 2.04]	3.67	0%	0.72
Jia et al. (25)	1.55 [1.04, 2.32]	3.59	0%	0.73
Kokotsakit et al. (29)	1.44 [0.99, 2.10]	4.40	0%	0.62
Mastroroberto et al. (28)	1.45 [1.00, 2.10]	4.40	0%	0.73
Puiu et al. (23)	1.15 [0.68, 1.93]	2.77	0%	0.84
Sabik et al. (21)	1.44 [0.96, 2.17]	4.40	0%	0.62
Schachner et al. (31)	1.45 [1.00, 2.10]	4.40	0%	0.73
Talwar et al. (24)	1.53 [1.04, 2.25]	2.91	0%	0.82
Yilik et al. (27)	1.44 [0.99, 2.09]	4.32	0%	0.63
All included studies	1.45 [1.00, 2.10]	4.40	0%	0.73

**Table 5:** Sensitivity analysis with leave-one out analysis for cannulation complication.

**Leave-one out analysis**	**OR, 95%CI**	**χ^2^ (Ch*i2*)**	***I*** **^2^**	***P*-value for heterogeneity:**
Do et al. (26)	3.16 [1.88, 5.29]	2.47	0%	0.48
Fleck et al. (30)	2.88 [1.70, 4.87]	1.94	0%	0.58
Jia et al. (25)	3.12 [1.87, 5.18]	2.54	0%	0.64
Kokotsakit et al. (29)	4.69 [1.95, 11.24]	1.26	0%	0.74
Mastroroberto et al. (28)	2.96 [1.67, 5.24]	2.32	0%	0.51
Puiu et al. (23)	3.12 [1.87, 5.18]	2.54	0%	0.64
Sabik et al. (21)	2.92 [1.74, 4.90]	1.20	0%	0.75
All included studies	3.12 [1.87, 5.18]	2.54	0%	0.64

## Discussion

Since 1970, cardiac surgeons has chosen axillary artery cannulation for cardiopulmonary bypass and it has received increasing attention for its advantage in preserving antegrade cerebral perfusion. In addition, compared with femoral artery cannulation with retrograde cerebral perfusion, it can decrease the incidence of cerebral embolization, retrograde dissection and malperfusion (33).

Axillary artery cannulation has been recommended as preferred cannulation site in type A aortic dissection in 2021 the American Association for thoracic surgery expert consensus (13) and 2014 ESC guideline (12). Axillary cannulation includes side-graft and direct cannulation. For side-graft cannulation sewing an 8–10 mm graft to axillary artery, with less cannula resistance and consequently decreased risk of peripheral damage (34). During circulatory arrest for aortic arch repair, the side-graft cannulation allowed unilateral antegrade cerebral perfusion by clamping the proximal innominate artery and indirect pressure monitoring by right radial artery catheterization. Moreover, it avoids the high-pressure oscillations that can occur as a consequence of constant antegrade selective cerebral flow during direct axillary artery cannulation, decreasing risk of neurological injury (10). For direct cannulation, it may occur vertebral artery malperfusion, increasing the risk of under-perfusion of the contralateral hemisphere. However, side-graft cannulation needs additional ~15–30 min and is technically demand (35). In addition, this technique may exacerbate further malperfusion or arch disruption when occurring dissection in the originating part of innominate or subclavian artery.

Moreover, some studies (21, 23, 27) have shown that side-graft cannulation has less neurological dysfunction and cannulation-related complications. In addition, the axillary artery may be fragile and small caliber (26), which is unsuitable for direct cannulation or transfer to side-graft cannulation when fail to direct cannulation. However, cardiac surgeons Ohria et al. (36) and Carino et al. (37) indicate that there are no differences between two cannulation methods, and direct cannulation can be less time consuming and simple.

Because different institutions have their own preference for axillary artery cannulation methods and the lack of randomized controlled trial has not resulting in reaching a consensus among cardiac surgeons. Thus, it is essential to select appropriate cohort studies to guide decision making to decrease potential complications and mortality for the high-risk cardiac surgery.

To the best of our knowledge, this is the first meta-analysis to compare the safety of direct axillary cannulation and side-graft axillary cannulation in patients undergoing cardiopulmonary bypass. A total of 1,543 patients suffering direct axillary cannulation or side-graft axillary cannulation retrieved from ten studies were included. The meta-analysis shows the advantage of side-graft cannulation over direct cannulation. We found that side-graft cannulation could effectively decrease the risk of neurological dysfunction and cannulation-related complications compared with direct cannulation. Although our subgroup analysis of thoracic aortic surgery showed no significiant difference in neurological dysfunction, direct cannulation has the potential to increase the incidence of neurological dysfunction from clinical perspective. Puiu et al. (23) has recently revealed that the higher incidence of neurological dysfunction from direct cannulation can be attributed to two potential reasons: (1) cerebral malperfusion owing to the coverage of vertebral-artery offspring; and (2) local dissection leading to cerebral malperfusion. Moreover, when putting cannulation into artery directly, it has the potential to damage the intima and has lower blood flow rates (38).

We also observed no difference in early mortality between the direct and side-graft cannulation groups. However, the results were inconclusive, as the incidence of early mortality was reported as a secondary result. However, the results were not conclusive as the incidence of early mortality was reported as a secondary outcome. This result should be treated with caution since most studies reported in-hospital mortality, and two studies reported 30-day mortality. We must consider this point as potential heterogeneity.

### Study Limitations

The following limitations distorting the results need to be consider. First, the results can be distorted by heterogeneity and confounding factors. This is because the two groups were unable to compare some factors, especially basic and procedure details. Moreover, Aortic pathologies, like acute aortic dissection and chronic aortic disease, are different. Pooled analysis of different aortic disease outcomes may distort the results of this study. Publication bias may also influence the analysis, as studies with negative results may not have been published or may have been overlooked. In addition, the included studies lack randomized controlled trials. The selection bias can also be due to the fact that the decision to use which cannulation methods was at the discretion of the cardiac surgeon.

Take into account the following for further research. First, the ten included cohort studies did not report long-term survival outcomes; Therefore, long-term results should be considered. paying more attention to some additional important clinical results, such as blood product requirements (23), the incidence of respiratory insufficiency and the incidence of sepsis, because Sabik et al. (21) reported that the most common comorbidities were respiratory insufficiency and sepsis, which could cause a poor prognosis and increase the therapy cost. Finally, to strengthen the evidence of meta-analysis, randomized control trials with well-performed and large-scale are need.

## Conclusion

This study suggests that side-graft axillary artery cannulation is a better strategy as it reduces the incidence of neurological dysfunction and cannulation-related complications. The superior outcome of side-graft cannulation can be ascribed to less damage to the artery intima and better cerebral perfusion, reducing the neurological dysfunction incidence. Nevertheless, the cannulation method for side-graft axillary cannulation should be chosen based on personal patient characteristics.

## Data Availability Statement

The original contributions presented in the study are included in the article/Supplementary Material, further inquiries can be directed to the corresponding author/s.

## Author Contributions

YX: conception and design. YX and PY: collection and assembly of data. YX, PY, and CL: data analysis and interpretation. YX, CL, and YL: manuscript writing. JH: read through and corrected the manuscript. All authors read and approved the final manuscript.

## Funding

This study was funded by the National Natural Science Foundation of China (No. 81670327), 1·3·5 project for disciplines of excellence, West China Hospital, Sichuan University (2020HXJS015), Sichuan Science and Technology Program (2019YJ0046), 1·3·5 project for disciplines of excellence-Clinical Research Incubation Project, West China Hospital, Sichuan University (No. 2019HXFH027) to research literature, statistical analysis, and the manuscript writing.

## Conflict of Interest

The authors declare that the research was conducted in the absence of any commercial or financial relationships that could be construed as a potential conflict of interest.

## Publisher's Note

All claims expressed in this article are solely those of the authors and do not necessarily represent those of their affiliated organizations, or those of the publisher, the editors and the reviewers. Any product that may be evaluated in this article, or claim that may be made by its manufacturer, is not guaranteed or endorsed by the publisher.
