# Supplementary figures and images for: Comparison of Post-operative Outcomes Between Direct Axillary Artery Cannulation and Side-Graft Axillary Artery Cannulation in Cardiac Surgery: A Systematic Review and Meta-Analysis
Source: Front Cardiovasc Med. 2022 Jun 10;9:925709. doi: 10.3389/fcvm.2022.925709 (PMC9226477; doi:10.3389/fcvm.2022.925709)

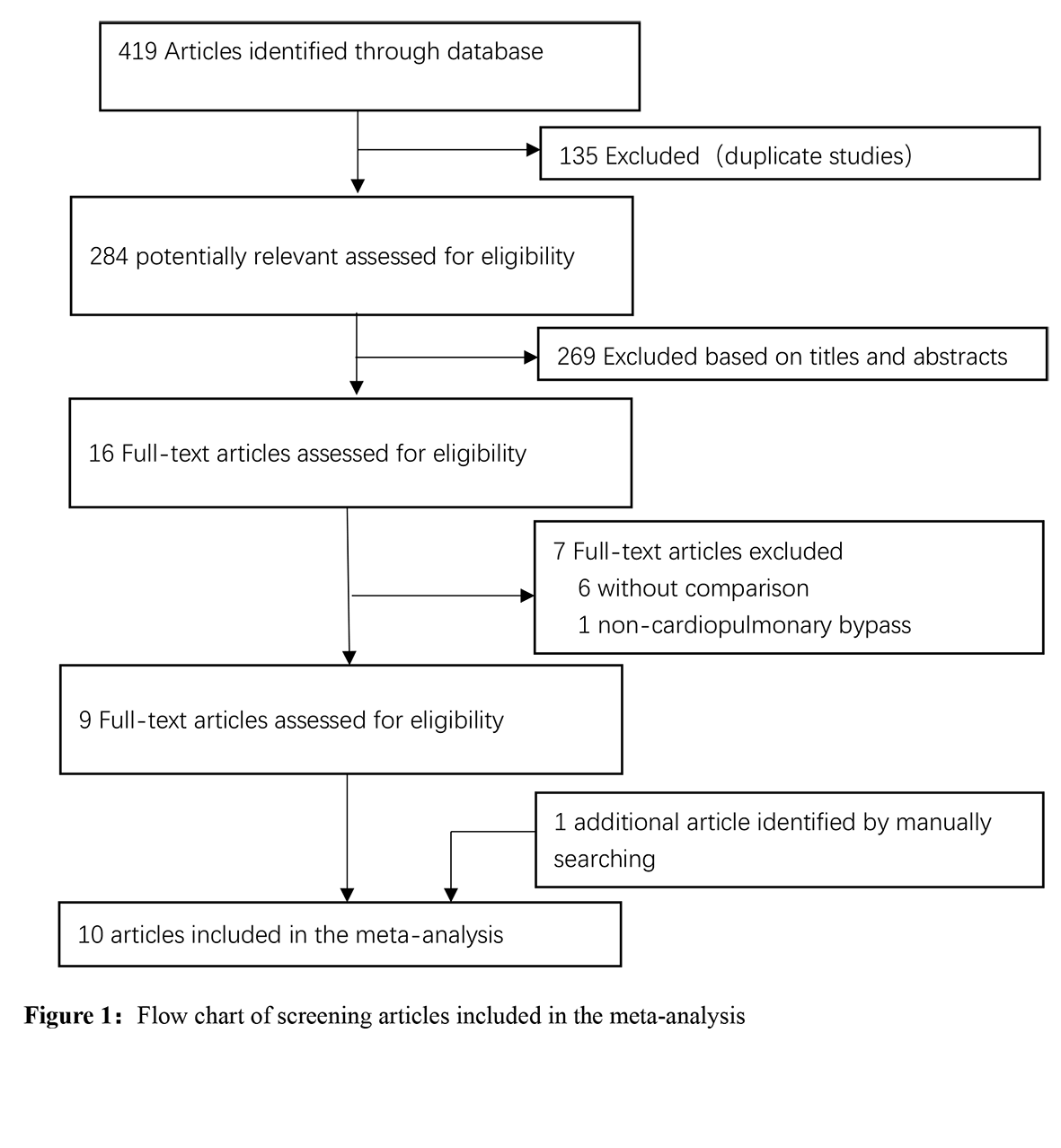

Supplement: Supplementary file 1 [file Data_Sheet_1.zip › supplementary material/figure 1.tif]

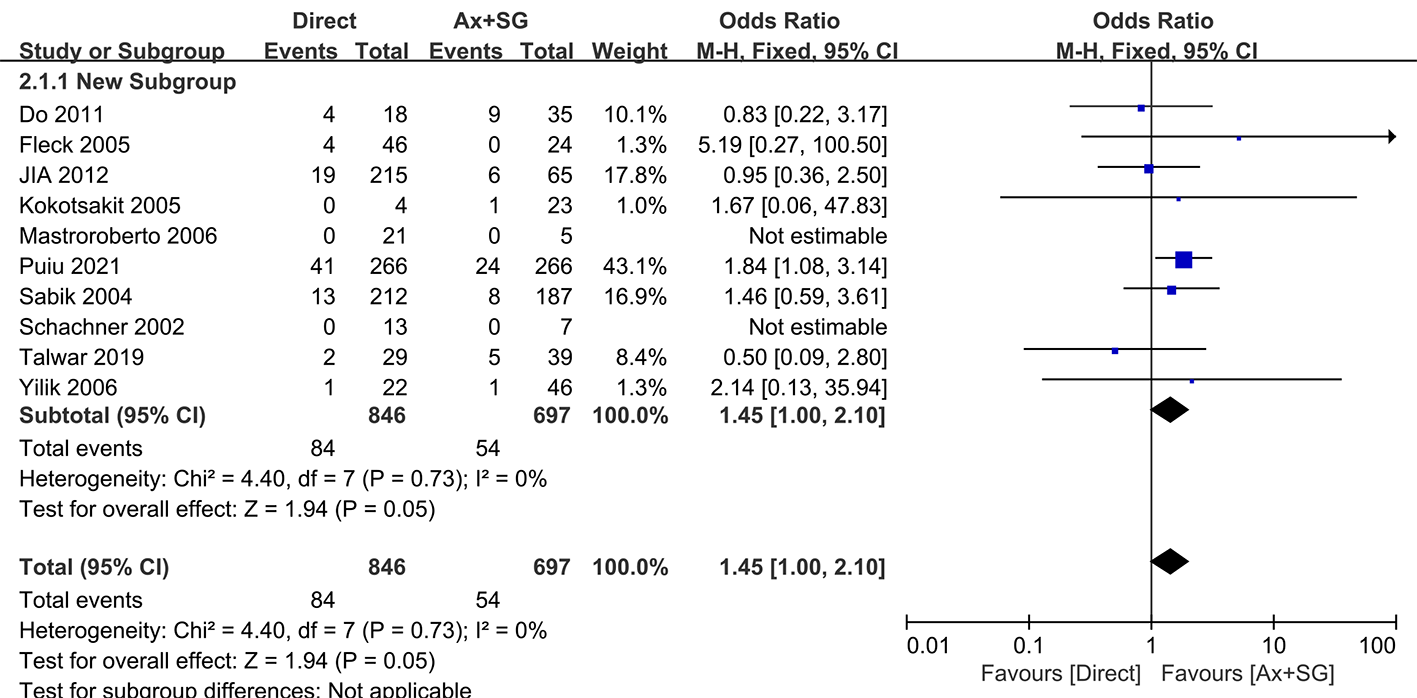

Supplement: Supplementary file 1 [file Data_Sheet_1.zip › supplementary material/figure 2.tif]

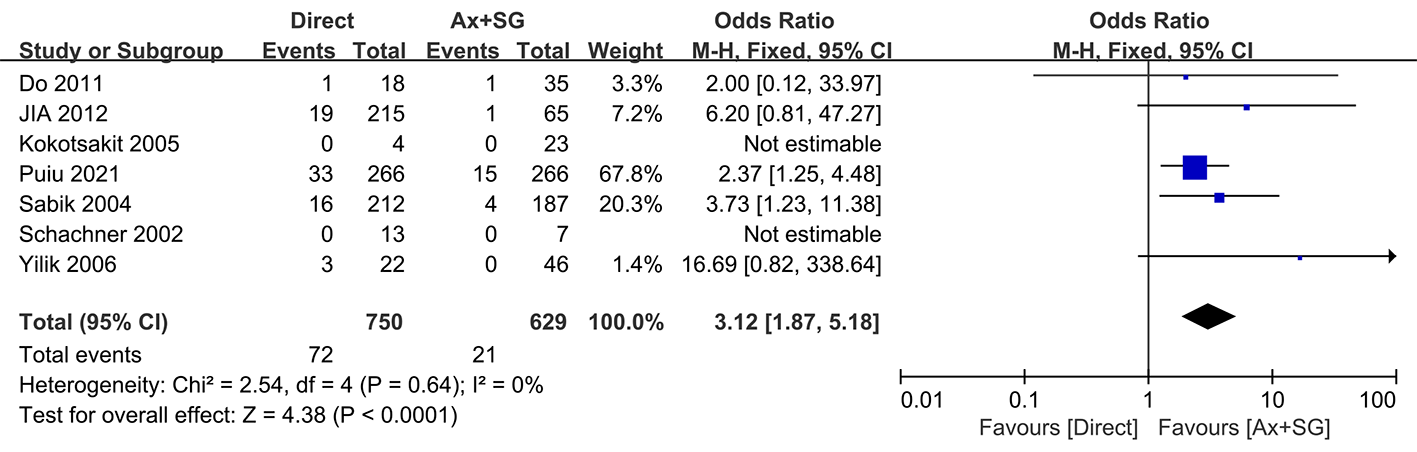

Supplement: Supplementary file 1 [file Data_Sheet_1.zip › supplementary material/Figure 3.tif]

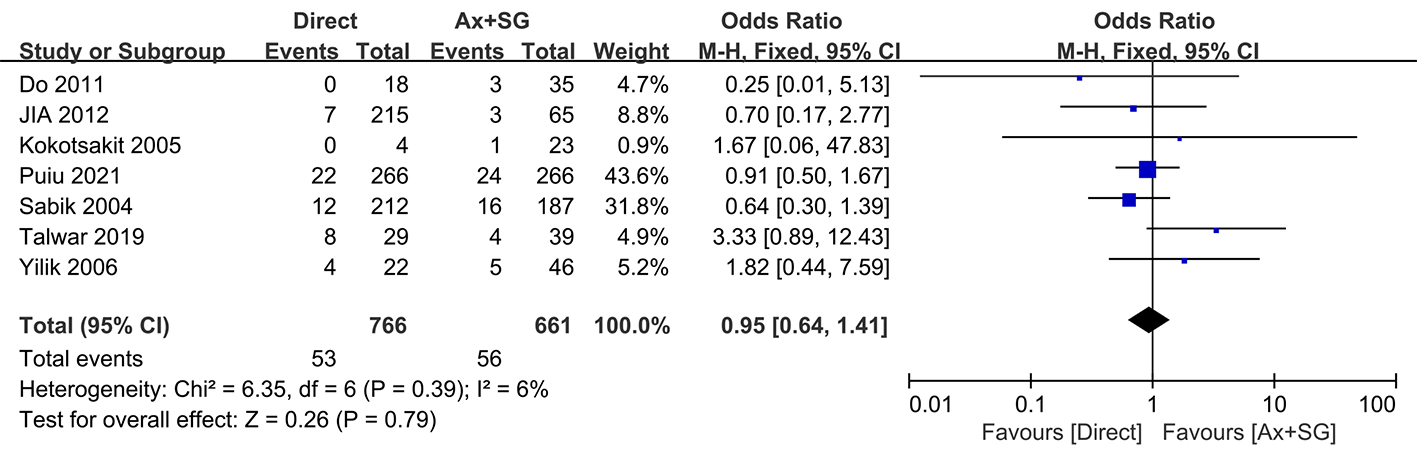

Supplement: Supplementary file 1 [file Data_Sheet_1.zip › supplementary material/Figure 4.tif]

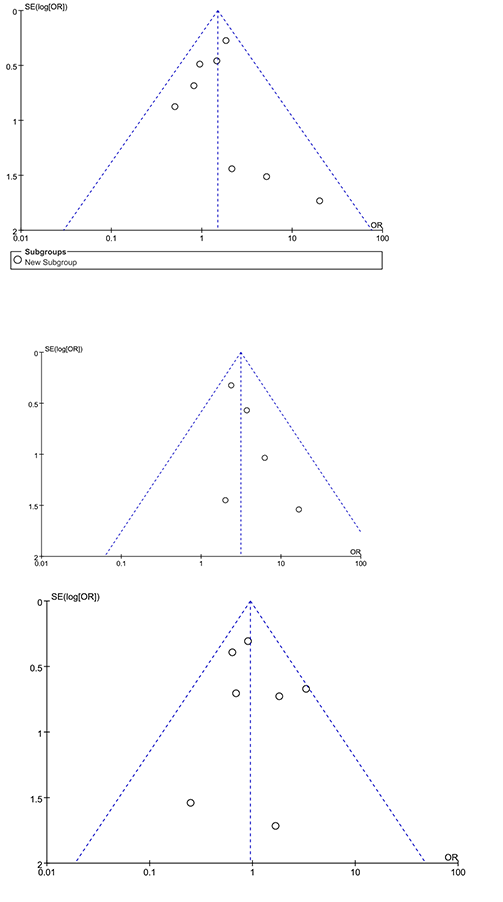

Supplement: Supplementary file 1 [file Data_Sheet_1.zip › supplementary material/figure 5.tif]
